# Soybean genetic resources contributing to sustainable protein production

**DOI:** 10.1007/s00122-022-04222-9

**Published:** 2022-10-14

**Authors:** Bingfu Guo, Liping Sun, Siqi Jiang, Honglei Ren, Rujian Sun, Zhongyan Wei, Huilong Hong, Xiaoyan Luan, Jun Wang, Xiaobo Wang, Donghe Xu, Wenbin Li, Changhong Guo, Li-Juan Qiu

**Affiliations:** 1 Key Laboratory of Molecular Cytogenetics and Genetic Breeding, College of Life Science and Technology, Harbin Normal University, Harbin, China; 2 Nanchang Branch of National Center of Oil crops Improvement, Jiangxi Province Key Laboratory of Oil crops Biology, Crops Research Institute of Jiangxi Academy of Agricultural Sciences, Nanchang, China; 3 The National Key Facility for Crop Gene Resources and Genetic Improvement (NFCRI) and MOA KeyLab of Soybean Biology (Beijing), Institute of Crop Science, Chinese Academy of Agricultural Sciences, Beijing, China; 4 School of Agronomy, Anhui Agricultural University, Hefei, China; 5 Soybean Research Institute, Heilongjiang Academy of Agricultural Sciences, Harbin, China; 6 College of Agriculture, Yangtze University, Jingzhou, China; 7 Biological Resources and Post-Harvest Division, Japan International Research Center for Agricultural Sciences, Tsukuba, Japan; 8 Soybean Research Institute, Key Laboratory of Soybean Biology of Chinese Education Ministry, Northeast Agriculture University, Harbin, China

## Abstract

**Key message:**

Genetic resources contributes to the sustainable protein production in soybean.

**Abstract:**

Soybean is an important crop for food, oil, and forage and is the main source of edible vegetable oil and vegetable protein. It plays an important role in maintaining balanced dietary nutrients for human health. The soybean protein content is a quantitative trait mainly controlled by gene additive effects and is usually negatively correlated with agronomic traits such as the oil content and yield. The selection of soybean varieties with high protein content and high yield to secure sustainable protein production is one of the difficulties in soybean breeding. The abundant genetic variation of soybean germplasm resources is the basis for overcoming the obstacles in breeding for soybean varieties with high yield and high protein content. Soybean has been cultivated for more than 5000 years and has spread from China to other parts of the world. The rich genetic resources play an important role in promoting the sustainable production of soybean protein worldwide. In this paper, the origin and spread of soybean and the current status of soybean production are reviewed; the genetic characteristics of soybean protein and the distribution of resources are expounded based on phenotypes; the discovery of soybean seed protein-related genes as well as transcriptomic, metabolomic, and proteomic studies in soybean are elaborated; the creation and utilization of high-protein germplasm resources are introduced; and the prospect of high-protein soybean breeding is described.

## Introduction

Soybean (*Glycine max* L.) originated in China and was known as “Shu” (Shu means legumes) in ancient China. It has a cultivation history longer than 5000 years. Having the largest planting area in the world among legume crops, soybean is the main source of edible vegetable oil and high-quality vegetable protein and provides a high-quality raw material for producing forage for livestock and aquatic animals (Kim et al. 2021a; Harada and Kaga 2019). Soybean protein is one of the high-quality vegetable proteins that have important health benefits. It is rich in eight essential amino acids, vitamins, flavonoids, and polysaccharides required by the human body (He and Chen 2013). Compared with cereal crops such as rice and maize, soybean protein has better features such as a high protein content, balanced amino acids, and excellent performance in emulsifying action and oil absorption, thereby being widely used in food processing, medicine, forage, and the chemical industry (Singh et al. 2007). Soybean-based products have become a daily nutritional solution for vegetarians and an important protein source for people on vegetarian and low-fat diets (Mortensen et al. 2009). In 1999, the important health benefits of soybean protein were recognized by the United States Food and Drug Administration (FDA) (Erdman 2000). In 2000, the United States Department of Agriculture approved access to soybean foods on university campuses, and two years later, tofu and soy yogurt were further approved as substitutes for animal meat and milk in school meal plans (He and Chen 2013), which strengthened the role of soybean products among the mainstream foods used in Western countries. The multiple demands for soybean products in the production of food, medicine, forage, and chemicals have greatly promoted the continuous increase in the global needs for soybean and the rapid development of the soybean industry. The yield and global production of soybean have continued to increase. The global production of soybean in 2021 was 382 million tons, an increase of 7.75% compared with that in 2020 and double the production (177 million tons) in 2000. The soybean protein produced in 2021 was approximately 153 million tons (https://baijiahao.baidu.com/).

Studies conducted by the World Health Organization (WHO)/Food and Agriculture Organization (FAO) (1985) have shown that soybean protein can provide all the essential amino acids for the balanced nutrition for human. Soybean protein is considered to be the high biological value protein among the plant-based proteins (García et al. 1998). The quality of soybean protein is comparable to animal proteins from meat, milk, and eggs (Millward 2012; Kudełka et al. 2021). Therefore, soybean protein is high-quality protein. With the increasing global population and the continuous improvement in per capita-based living standards, people have a more comprehensive understanding of the nutritional value and health benefits of soybean protein. In particular, under the impact of the COVID-19 pandemic, the role of soybean has become more prominent in securing the supply of food and dietary nutrients. The acceptance and need for high-quality soybean products in many countries and regions have increased significantly. According to an estimation with incomplete data, more than 12,000 food recipes worldwide require soybean protein as the raw material, which largely meets people's needs for vegetable protein. In addition, soybean meal has become the important source of high-quality protein for feed industry (Banaszkiewicz 2011; He and Chen 2013). Therefore, effectively securing the sustainable supply of soybean protein has become a major focus in soybean production and research, which can be achieved through breeding for high-yield and high-protein varieties using the rich genetic resources of soybean. This paper systematically reviews the origin, production, and development of soybean as well as the utilization of genetic resources in the breeding of high-protein soybean varieties.

## Nutritional and economic value of soybean protein

### Soybean protein is a complete vegetable protein

**Fig. 1:**
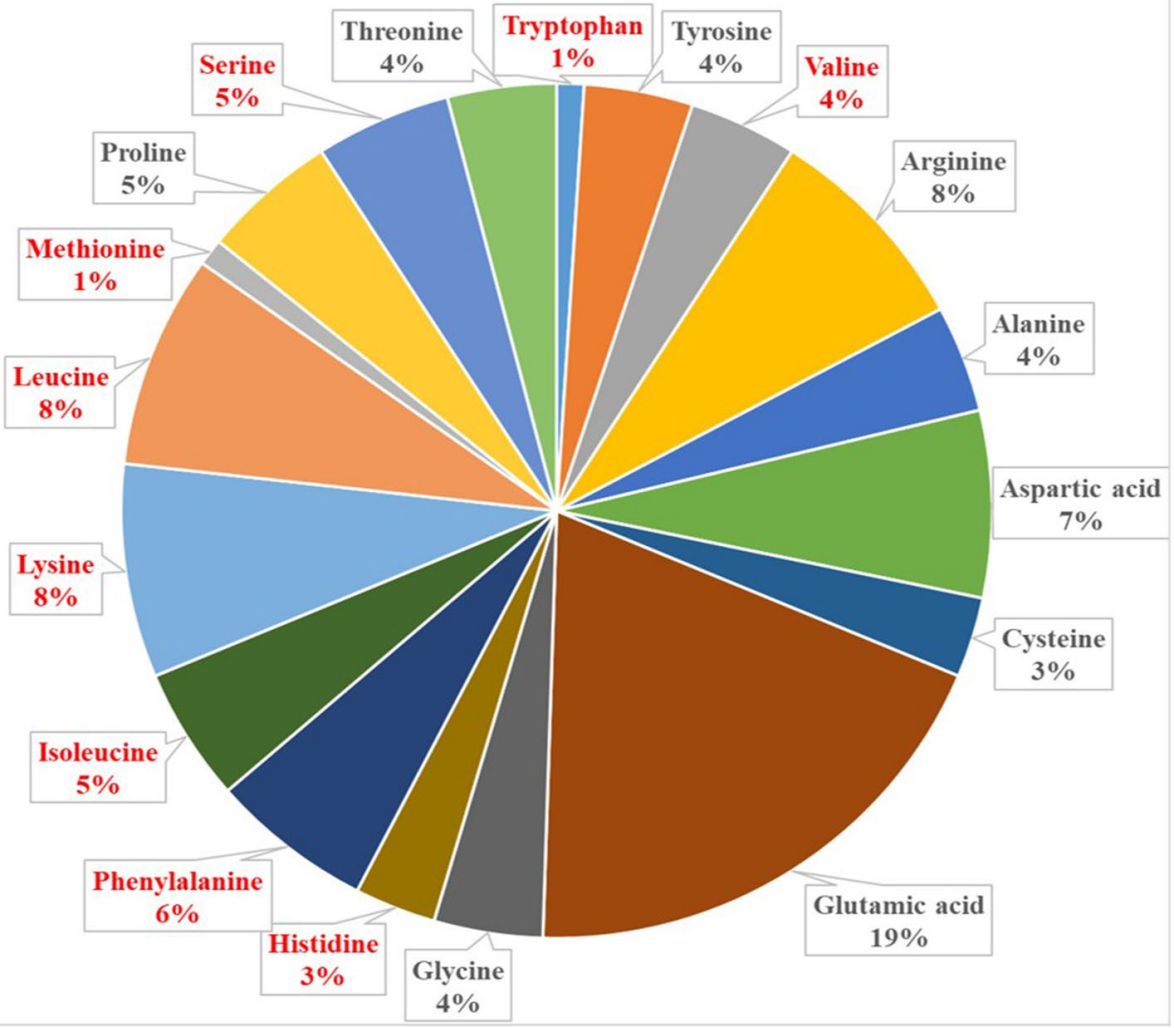
The amino acid composition of soybean protein. The red grid represents essential amino acid, while the black grid represents non-essential amino acid

Oil and protein are the important commercial interest in soybean. Soybean seeds have the high protein content of about 40% and oil content of about 20%, respectively (Banaszkiewicz 2011). Based on 2018–2019 data from the United States Department of Agriculture (USDA), 87%, 6% and 7% of the global soybean output are used for soy oil and soy cake, foods for human consumption, and whole-bean animal feed, respectively (https://apps.fas.usda.gov/psdonline/app/index.html#/app/advQuery). Soybean-based food products are a source of high-quality vegetable protein, such as tofu, soy milk, and yuba (Rizzo and Baroni 2018). Globulin is largely responsible for the nutritional value of soybean meal, accounting for about 70% of the total protein in soybean seed (Krishnan et al. 2000; Kudełka et al. 2021). The amino acid composition of soybean protein is similar to that of animal protein (Kudełka et al. 2021). Soybean protein is also rich in eight essential amino acids required by the human body and is recognized as a complete protein source that also contains histidine, which is not produced by infants (Chen et al. 2012; Kim et al. 2021a). The percent rates of nine amino acids contents are displayed in Fig. 1, including histidine, phenylalanine, methionine, serine, valine, isoleucine, leucine, tryptophan, and lysine. According to the Protein digestibility-corrected amino acid scores (PDCAAS), soybean protein ranks first among vegetable proteins and is comparable to that of milk and egg proteins (https://foodproteins.globalfoodforums.com/food-protein-articles/soy-protein-delivers-on-nutrition-quality-sustainability/). Compared to cow’s milk, soy milk is free of lactose and cholesterol (He and Chen 2013).

Soybean protein also benefits human health and prevents multiple diseases (Asif and Acharya 2013). In 1999, the FDA pointed out that the daily intake of 25 g of soybean protein may reduce the risk of heart disease (Erdman 2000). Soybean protein consumption may significantly reduce total cholesterol, LDL (low-density lipoprotein) cholesterol, and triglyceride concentrations in serum, thereby reducing the risk of coronary heart disease (Anderson et al. 1995; Zhang et al. 2003). Multiple studies have shown that the consumption of soybean protein may reduce the risk of breast and prostate cancer, benefit the kidneys, alleviate diabetes, prevent osteoporosis, lower blood pressure, relieve depression, and improve menopausal symptoms (Singh et al. 2007; Jayachandran and Xu 2019). The use of soybean protein may also inhibit fat accumulation, increase fat metabolism, and effectively regulate the expression of appetite suppressors, thereby contributing to weight reduction and obesity prevention (Messina 2016; Ramdath et al. 2017).

### Soybean protein plays an important role in the food, forage, and chemical industries

Soybean is used to produce food products, food additives and industrial additives (Modgil and Kumar 2021; Kudełka et al. 2021). Soybean has been used as a basic ingredient in traditional dishes since ancient times and is the main source of high-quality vegetable protein for humans (Kim and Kwon 2001). Fermented and non-fermented foods are two types of processed foods based on soybean. Non-fermented soybean foods mainly include edamame, soybean sprouts, dehydrated soybeans, soybean flour, soy milk, tofu, bean skin, and yuba. Fermented soybean foods include soy sauce, fermented soybeans, natto, miso, soybean paste, fermented bean curd, and fermented soy milk (Kada et al. 2008; He and Chen 2013; Harada and Kaga 2019; Jayachandran and Xu 2019). In addition to traditional processing, soybean is also widely used in the production of functional foods, such as infant formula soybean milk powder, protein powder, artificial meat, dairy-free milk alternatives, and plant-based foods (Andres et al. 2012; Rizzo and Baroni 2018). Soybean-based products are also used as food additives to improve taste, increase the elasticity and oil- and water-holding capacity of foods, and enhance the storability of foods (Singh et al. 2007).

In the field of forage processing, soybean meal is the high-quality vegetable protein source considering its quantity and quality (Banaszkiewicz 2011). According to European Feed Manufacturers Federation (FEFAC) in 2007, about 18.6% edible oil and 78.7% protein-rich meal are produced in soybean after oilseed processing. The protein content in soybean meal is as high as 40–49%, and the soybean meal is mainly used for animal nutrition on the feed market (Banaszkiewicz 2011). 85% of soybean meal produced globally is used for forage production for non-ruminant animals such as poultry and pigs (Mili et al. 2012). The global production of soybean meal continues to increase. In 2019, the global production of soybean meal was 243 million tons (USDA, https://www.fas.usda.gov/), and in 2021, it increased to 251 million tons. It is predicted that the global production of soybean meal in 2022 will reach a new record of 256 million tons (https://www.feedtrade.com.cn/sbm/stat/2160577.html). The protein in soybean meal has become the most important source of protein not only in livestock forage but also in aquaculture feed. Except for methionine, the amino acid composition of soybean meal is similar to that of fish protein powder and can fully meet the amino acids needs of fish farming (INRA 2004). Due to its high quality and low price, soybean meal has gradually replaced expensive fish protein powder and is playing an increasingly important role in aquaculture (Yun et al. 2018). Soybean protein concentrate (SPC) can be used to largely replace skimmed milk powder and milk for feeding calves and can also be used as a pre-fermented feed to replace skimmed milk powder, whey protein, and fish protein powder for piglets (Dei. 2011). In the chemical industry, the widespread use of polyethylene, polypropylene, and polystyrene produced from petroleum causes serious white pollution and energy crises. Soybean protein can be sustainably reproduced at a low cost and can be an effective substitute for petroleum in some areas of the chemical industry (Tian et al. 2018a). In recent years, the problem of environmental pollution has attracted increasing attention, and the regulations have become more and more strict. How to effectively solve the problem of white pollution is a serious concern of the public. Soybean protein-based biodegradable materials are widely used in the chemical industry and play an increasingly important role in the production of plastics, foams, edible films, nanofibers, adhesives, novel composite materials, and biomedical substances, which in turn increase of the output and economic value of soybean and soybean protein (Liu et al. 2013; Luo et al. 2015; Thakur et al. 2015; Tian et al. 2018b).

## The origin of soybean and current situation of soybean production

### The origin and spread of soybean

It is believed that soybean has a long history of domestication in China. Cultivated soybean (*Glycine max* (L.) Merr.) and wild soybean (*Glycine soja* Sieb. et Zucc.) are two distinct species in soybean. Wild soybean is the original ancestral species of cultivated soybean and is an important germplasm resource for high-protein breeding due to its high protein content. Wild soybean occurs mainly in China, North Korea, Japan, and Russia and has the widest distribution in China (Li 1988; Kuroda et al. 2008). Cultivated soybean originated from the long-term artificial domestication and selection of wild soybean (Tian and Gai 2001) and has a cultivation history longer than 5000 years. Soybean was first introduced from China to neighboring countries Korea and Japan. In 1737, soybean was introduced into France and then into Europe. In 1765, soybean was introduced into the USA. In 1882, soybean was introduced into Argentina and began to spread in South America. In 1898, soybean was introduced from northeastern China into central and northern Russia. In 1950, Brazilians began to grow soybean to improve soil fertility, and the planting area in Brazil gradually expanded thereafter. Brazil became the world's largest soybean producer in 2021. Soybean is currently planted worldwide. Numerous soybean varieties have been developed to adapt to different ecological conditions and meet the diverse needs of multi-dietary culture (Chang 1989; Abe et al. 2003; Hymowitz and Shurtleff 2005; Patil et al. 2017).

**Fig. 2:**
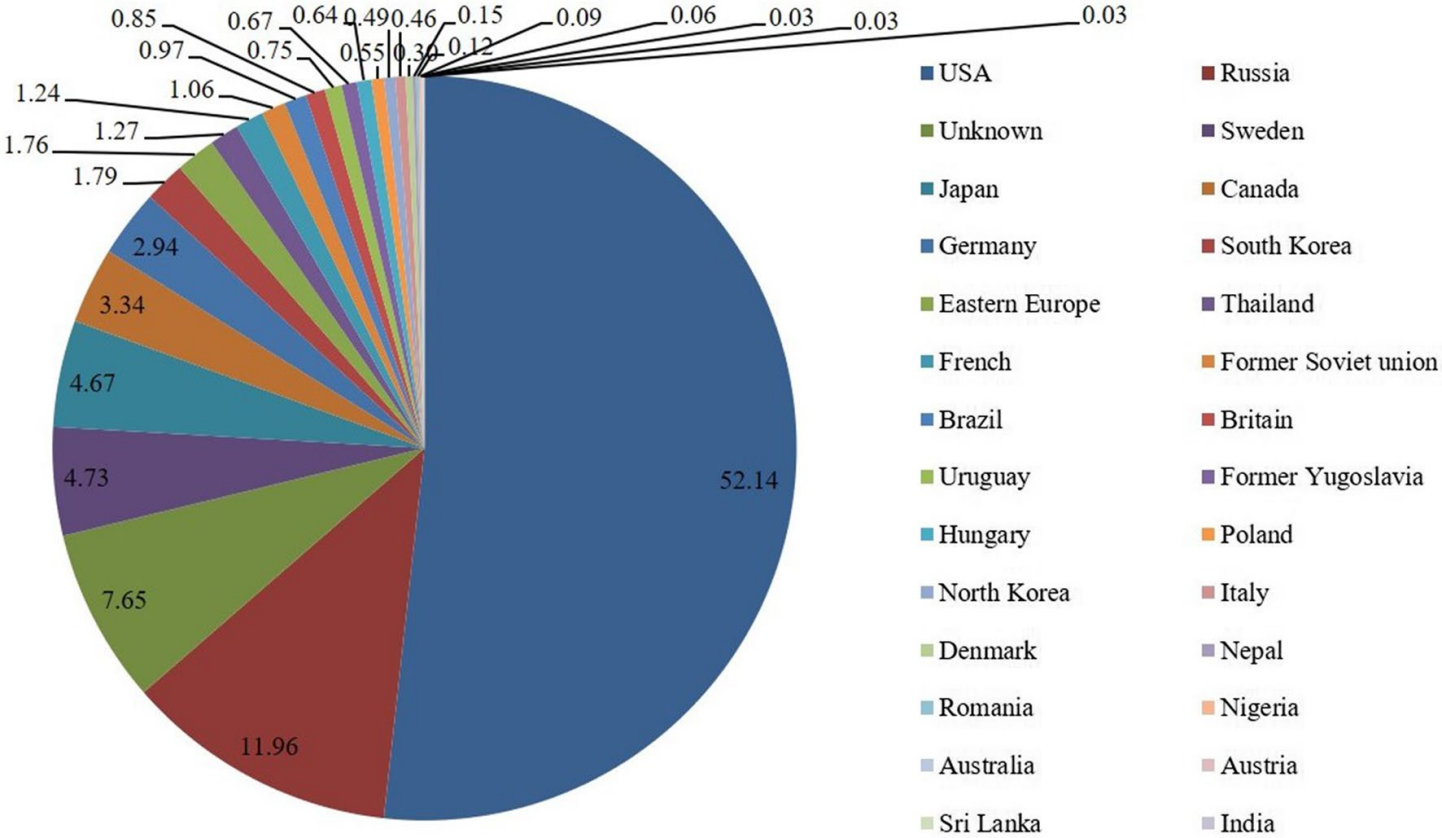
The number and ratio of soybean germplasm collected from different sources in the Chinese Crop Germplasm Resources Bank

Soybean originated in China where there are very rich soybean germplasm resources with useful characteristics. The Chinese Crop Germplasm Resources Bank has collected and preserves more than 33,000 soybean germplasms from different sources (Fig. 2). The soybean germplasm resources that spread from China have promoted the breeding of new soybean varieties around the world, making important contributions to the development of the soybean industry and the sustainable production of protein worldwide. The early soybean varietal resources in the USA were mainly collected from China and its surrounding areas. Dorsett collected 1,500 soybean resources from northeastern China in 1924–1926, subsequently, Dorseet and Morse collected about 4,500 soybean resources from China, Japan, and the Korean peninsula from 1929 to 1931 (Chang 1989; Singh and Hymowitz 1999) and screened varieties such as Blackeye (introduced from Heilongjiang Province, China), Cayuga (introduced from Heilongjiang Province, China), Aksarben (introduced from Liaoning Province, China), and Barchet (introduced from Hebei Province, China) for soybean production in North America (Bernard et al. 1988). In the middle of the twentieth century, the outbreak of cyst nematode disease seriously threatened the soybean industry of the United States. The cyst nematode-resistant germplasm Peking screened from Chinese soybean resources was used in soybean breeding in the USA, and soybean varieties such as Pickett, Dyer and Bedford were successfully bred. These varieties effectively helped control the occurrence and spread of cyst nematode disease after they were used in production, and as a result, they rescued the soybean industry of the United States. Peking is still one of the main resources of resistance genes to cyst nematode among the varieties used in soybean production in the USA (Qiu et al. 2006). In the past few decades, soybean genetic resources collected from northern China have effectively promoted the breeding of new soybean varieties and the development of the soybean industry in the northern United States and Canada. The soybean genetic resources collected from southern China became the ancestral species of the varieties bred in the south of the United States (Chang 1989; Bernard et al. 1988). North American soybean varieties retain 72% of the sequence diversity of Asian landraces but have lost 79% of rare alleles and have a serious population bottleneck (Hyten et al. 2006), which is due to the few germplasms introductions from China and its surrounding areas when the breeding was conducted. Bandillo et al. (2015) conducted a microarray analysis of 14,000 soybean germplasm accessions collected by the USDA and found that 59% of the soybean resources in the USA have a Chinese origin; 49% of the American soybean ancestors have homology with Chinese soybean genes, verifying the fact that soybean originated in China and has benefited the world.

Wysmierski and Vello (2013) identified 60 ancestral parents from 444 Brazilian soybean varieties, of which 55.3% of the genetic basis was contributed by CNS (PI 548445), S-100 (PI 548488), Roanoke (PI 548485), and Tokyo (PI 548493). Among them, CNS, S-100, and Roanoke were from China. Zhou et al. (2000) identified 74 ancestral parents among 86 soybean varieties registered in Japan from 1950 to 1988, of which 76% of the genetic basis was contributed by Japanese ancestral parents, and 2%, 5%, and 2% were contributed by foreign ancestral parents from North America (the United States and Canada), China, and Korea, respectively. The long history of soybean breeding and the geographical isolation of Japan account for the diverse genetic basis of Japanese soybean varieties. The 1,300 soybean varieties bred in China from 1923 to 2005 were derived from 670 ancestral parents, of which 113 were core parents with a large contribution to breeding and 20 were foreign resources that accounted for 17.7% of the parents (Xiong et al. 2007). In recent years, soybean varieties with high protein content and high quality such as Huachun 6 (45.80%), Huaxia 4 (46.15%), and Tongnong 10 (46.22%) bred in China include the genes from elite foreign germplasms such as Brazil 8 and Tokachi nagaha (Ouyang et al. 2009; Cui et al. 1999; https://www.chinaseed114.com/). In particular, Tokachi nagaha is one of the most widely used imported resources in soybean breeding in China. As of 2005, 195 soybean varieties had been bred, which are important backbone parents for spring soybean breeding in northern and northeastern China and have a genetic contribution rate of 0.78–50% to the new varieties (Guo et al. 2007). These studies show that the use of foreign soybean resources to broaden the genetic basis of cultivated soybean is one of the common characteristics of soybean breeding worldwide. The spread of Chinese soybean germplasm to the rest of the world has promoted the sustainable production of protein on a global scale, and the use of foreign soybean germplasm resources has effectively secured the supply of edible vegetable protein in China.

### Current status of global soybean production

Before the 1950s, China was the largest soybean producer in the world. After World War II, the USA first strengthened the development and utilization of soybean varieties, and in 1954, soybean production in the USA began to surpass that in China and ranked first in the world. In the USA, soybean output has increased from 18.47 million tons in 1961 to 120 million tons in 2021, i.e., an increase of 6.5 times. Brazil is an emerging market in global soybean development and an important engine for pushing the continuous growth of global soybean production. The Brazilian soybean industry has the characteristics of a late start, rapid development, and great growth potential. Since 1974, the total annual soybean output in Brazil has surpassed that in China, and its global output share has increased sharply from 1.01% in 1961 to 36.74% in 2020. In 2021, the soybean output in Brazil was about 144 million tons, which surpassed the 120 million tons in the United States, and Brazil became the largest soybean producer in the world (https://www.fas.usda.gov/). Argentina is the third largest soybean producer and its soybean output has also grown rapidly. Soybean production in Argentina has increased from 1000 tons in 1961 to 50 million tons in 2020, accounting for 13.81% of the market share. The total annual soybean output in Argentina in 2021 was estimated to be 49.5 million tons (https://www.fas.usda.gov/). China is the fourth largest soybean producer in the world, but the average annual output is limited. The total annual output increased from 6.21 million tons in 1961 to 19.6 million tons in 2020, which is an increase of 3.16 times. However, the proportion of global output decreased from 23.1% in 1961 to 5.41% in 2020, and now China has become the world's largest soybean importer because of the large population, and the limited arable land needs to be preferentially used for producing rice and other main food crops (Hou et al. 2010; Gu et al. 2014; Kim et al. 2021b; Qiu et al. 2006; USDA, https://www.fas.usda.gov/).

The major soybean producing countries mainly focused in the USA, Brazil, Argentina, China and India, accounting for about 85% of the world’s soybean production (Gupta and Manjaya 2022). Considering their total soybean output, these countries will provide some good prospects for increasing sustainable soybean production. According to statistics, the United States accounts for about 40% of the annual exports to China (http://www.amis-outlook.org/news/detail/en/c/1144134/). With the increasing expansion of soybean export, soybean becomes the greatest cultivated crop than wheat and corn in the United States (Nosowitz 2017). In 2017, Brazil (USD 26.1 billion), the United States (USD 22.8 billion), and Argentina (USD 3 billion) were the largest soybean-exporting countries by total value, but China (USD 38.1 billion) was the largest importing countries (https://resourcetrade.earth/). Nevertheless, the global trade flow is largely reshaped in soybean. The current United States–China trade war has a direct effect on soybean industry that soybean export from the United States will likely be turned to other markets, and Brazil, Argentina and Ukraine will become more attractive markets for soybean purchases in China (http://www.amis-outlook.org/news/detail/en/c/1144134/). In 2019, China accounts for around 80% of Brazil’s soybean exports (https://www.hellenicshippingnews.com/brazil-soybeans-lose-protein-china-sales-at-risk/). The soybean production has not been interrupted by the impact of COVID-19 in the main producing countries (https://ussoy.org/u-s-soy-industry-strives-to-maintain-export-channels-supply-chain-of-high-quality-soy-amidst-covid-19-concerns/). Moreover, climate change also has a strong effect on soybean production. The World Bank estimates that soybean yields in Brazil could drop by 30% or more by 2050, but less drop in Argentina (Fernandes et al. 2012). According to the published report from the EU Commission, the soybean planting area in the 27 EU countries has more than doubled over the past 10 years and was almost 958,000 hectares in 2021 (https://www.biobased-diesel.com/post/soybean-area-in-eu-is-growing). In 2021, Italy (286,000 hectares), France (172,000 hectares), Romanian (160,000 hectares), Croatia (85,000 hectares) and Austria (76,000 hectares) were the five largest EU soybean producer. Europe produced about 10 million tons of traditional soybean per year, while genetically modified soybean imports accounted for more than 80% of plant protein, and 90–95% of that was used for animal feed (https://insights.figlobal.com/plant-based/european-soy-quest-protein-self-sufficiency).

Meanwhile, the versatile consumption demand for soybean is the major factor in maintaining the soybean production growth. In addition, the continuous intensification of the contradiction between population expansion and resource shortages as well as the increase in people's living standard have led to an increase in the global demand for soybean, which has further promoted the rapid development of the soybean industry and has caused a change in the market share of soybean producers. Soybean varieties with high yields and high protein contents will play a very important role in maintaining human health and sustainable social development.

## Phenomics of soybean protein

### Genetic characteristics of soybean protein

Soybean seed protein content is a quantitative trait determined mainly by the additive effects of multiple genes that interact with each other. This trait has a complex genetic mechanism and can be affected by the interaction between genotype and the environment (Pathan et al. 2013). The protein content of soybean seed is often negatively correlated with the oil content because the synthesis of protein and that of oil compete for limited carbon and energy supplies. The soybean seed protein content is also negatively correlated with agronomic traits including the grain yield, plant height, number of nodes and branches on the main stem, the flowering time, and maturity date (Dhungana et al. 2017; Chung et al. 2003; Cober and Voldeng 2000; Phansak et al. 2016). These negative correlations are main obstacles to the breeding of soybean varieties with high yield, high oil content and high protein content. In addition, the protein content of soybean seeds varies significantly among genotypes and can be affected by environmental conditions, planting location, sowing date, water and fertilizer conditions, and temperature (Li et al. 2004a, b). The database of soybean germplasm resources showed that seed protein contents of wild soybean and cultivated soybean are 35.5–56.9% and 31.7–57.9%, respectively (https://npgsweb.ars-grin.gov/). These wide variations will provide great potentiality for genetic improvement of protein content (Wang et al. 2021a).

### Distribution of wild soybean germplasm resources in terms of the protein content

Soybean originated in China where there are the most abundant wild soybean resources. More than 7000 wild soybean resources were collected and are preserved in the China National Crop Gene Bank, accounting for about 90% of the wild soybean resources worldwide (Dong 2008). Wild soybean has rich genetic diversity and resistance to diseases and stresses as well as a higher protein content (46–48%) than that (38–42%) of cultivated soybean (Patil et al. 2018). Therefore, wild soybean can be used in the genetic improvement of soybean, especially the breeding of varieties with high protein content (Wang et al. 1998; Hu et al. 2011; Xue et al. 2014; Li et al. 2017). Wee et al. (2018) analyzed 334 Japanese wild soybeans and found that their average protein content was 54% (48.6–57.0%). Li (1993) found that the average protein content of more than 5200 wild soybean accessions collected in China was 44.99%, and their protein content showed a distribution pattern of high in the south and low in the north. In addition, Wang et al. (1998) determined the protein content of 6115 wild soybean resources and found that their average content was 45.36%, which is 1.05% and 2.06% higher than that of cultivated soybean collected from China and foreign countries, respectively. These studies further show that wild soybeans are characterized by a high protein content.

### Distribution of cultivated soybean germplasm resources in terms of the protein content

There is an inverse relationship between the protein content of cultivated soybean and latitude; the lower the latitude, the higher the protein content (Wang et al. 1998, Rotundo et al. 2017). The protein content of a particular variety varies significantly under different growing conditions. Cv. Williams was planted in 186 locations in 76 countries and regions around the world, and its average, lowest, and highest protein contents were 42.53%, 32.8%, and 48.7%, respectively (Li et al. 2004a, b). The protein content of soybean grown in the southern United States is generally higher than that grown in the northern US (Rotundo et al. 2017). In China, the protein content of soybean grown in the Huanghuai region and southern multiple cropping areas is higher than that grown in Northeast China, which is the main soybean producing area (Liu et al. 2017). Wang et al. (1998) reported that the protein content of cultivated soybean collected and preserved in China was an average of 2.06% higher than that of soybean introduced from abroad (Wang et al. 1998). The sowing season and sowing date also significantly affect the soybean protein content. Spring sowing is favorable for oil biosynthesis, while autumn sowing and short-day conditions are favorable for protein accumulation. In addition, delayed sowing may result in a decrease in the soybean protein content.

There are abundant genetic variations in the protein content among diverse soybean germplasm resources. An analysis of soybean germplasm collected and preserved by the Chinese Crop Germplasm Resource Bank showed that the average protein content of Chinese soybean germplasm was 42.47%, whereas that of foreign soybean germplasm was 41.23% (http://crop.agridata.cn). Grieshop and Fahey (2001) analyzed the protein content of 48 Brazilian soybean accessions, 49 Chinese soybean accessions, and 36 American soybean accessions and found that the protein content of Chinese soybean (42.14%) was significantly higher than that of Brazilian (40.86%) and American (41.58%) soybeans. A study of soybean germplasm encompassing maturity groups from 00 to VIII in the USA indicated that the protein content was higher in the south (41.1%) than in the north (40.7%) (Yaklich et al. 2002). Shi et al. (2010) analyzed 105 soybean accessions from the United States, Japan, and Korea and found that their average protein content was 42.9% and that of United States accessions was 41.3%. In addition, Lee et al. (2021) found that the average protein content of soybeans from South Korea, North Korea, Japan, the USA, and Russia was 39.7%, 39.2%, 38.8%, 38.0% and 37.2%, respectively. In Europe, the soybean varieties are mainly from MG 0 to MG II in Italy, while the soybean varieties are mainly from MG 000 to MG II in France (Rüdelsheim and Smets 2012). Soybean cultivation had great potential for European agriculture, but adaptation was the central issue for soybean sustainable development in Europe (Kurasch et al. 2017a). Kurasch et al. (2017a) revealed that desirable varieties by breeding improvement might be the prerequisite for expansion of soybean cultivation in Europe, and the protein content of 1008 RILs from an early European soybean variety is about 39–42% or above 42%. In China, the soybean varieties are mainly bred for food; therefore, the protein content of Chinese soybean germplasm is higher than that of foreign counterparts. Wang et al. (1998) analyzed the protein content of 21,050 soybean accessions collected from several provinces in China and found that their average protein content was 44.31%, and the average protein content of the accessions collected from individual provinces was 42.89% (Heilongjiang), 42.24% (Jilin), 43.30% (Liaoning), 45.05% (Jiangsu), 44.35% (Anhui), 47.10% (Jiangxi), 46.03% (Hunan), 46.17% (Guizhou), and 45.27% (Fujian). These results showed that soybean protein content display the geographic distribution variation in seed protein content.

The variability of seed composition such as protein, oil and fatty acid concentrations may be influenced by genotype, environment, management practices and their interactions (Bellaloui et al. 2011). Different latitude regions show environmental fluctuations especially in photoperiod and temperature. Soybean is a short-day plant, and its photo- and thermo-sensitivities seriously restrict the growing season and geographic distribution (Szczerba et al. 2021). Elevated temperature (> 28 °C) and shortened day length may contribute to maintaining high protein content by increasing the rate of nitrogen translocation to the seed and seed growth rate (Cure et al. 1982; Dornbos and Mullen 1992; Gibson and Mullen 1996). In 2003–2004, soybean seed showed significant differences on the content of crude protein, essential amino acids and non-essential amino acids in different Brazilian states as the diversity of growing conditions, including climatic changes, topography, and soil fertility (Goldflus et al. 2006). Due to the warmer weather and longer days, the same genetically modified soybean had higher protein content in Brazil (about 37%) than United States (about 14.1%) in 2017 (https://geneticliteracyproject.org/2018/01/30/brazils-higher-protein-gmo-soybeans-hurting-us-exports-china/). Rotundo et al. (2009) reported that high protein varieties have greater assimilate availability per seed during seed-filling period, and higher protein levels in high yielding varieties can be achieved with greater sucrose and nitrogen assimilate supply per seed. Additionally, maintaining adequate soil moisture during the reproductive stage is critical for the accumulation of protein content in soybean seed (Wijewardana et al. 2019). Moreover, UV-B radiation can cause significant alteration that protein content decline linearly with higher doses of UV-B radiation (Reddy et al. 2016). Therefore, different environmental conditions during pod-filling period may have a considerable effect on soybean protein content.

## Soybean protein-related genomics

### Soybean protein content-related genes

A high protein content is an important goal of soybean quality breeding. The cloning of soybean protein content-related genes/quantitative trait loci (QTL) is helpful for breeding new soybean varieties with high protein content and high yield using molecular technology. Several researchers have used a variety of materials and methods to study the genes/loci related to the soybean protein content. Existing studies have identified the QTLs related to the soybean protein content on 20 soybean chromosomes, and related new QTLs are still being reported (Bandillo et al. 2015; Kim et al. 2016; Lee et al. 2019; Wang et al. 2020a). For example, QTLs *cqProt-001* and *cqProt-003* were first identified on chromosomes 15 and 20 (Diers et al. 1992), QTL *Satt127* and *Iasu-A144H-1* were detected in wild soybean to have an association with the protein content (Sebolt et al. 2000), QTLs have been mapped on the B1 and L linkage groups (Chapman et al. 2003), the protein content-related QTLs *Seed protein 34–10*, *Seed protein 36–31*, and *Seed protein 36*–*32* were identified on chromosome 19 (Lu et al. 2013; Mao et al. 2013), Kim et al. (2016) identified a high protein and low oil content QTL on chromosome 15 in PI 407788A, and Warrington et al. (2015) identified a major QTL associated with the protein and amino acid contents on chromosome 20. According to data in the Soybase database, 249 QTLs related to the soybean protein content have been identified (https://soybase.org/), mostly in the linkage group I (chromosome 20), explaining 7–65% of the observed phenotypic variations, followed by those detected in linkage group E (chromosome 15), linkage group C2 (chromosome 6), and linkage group G (chromosome 18) (Table 1).

**Table 1:** Information of QTLs for protein content in soybean

Chr	Linkage Group	Numbers of QTL	Genetic contribution (%)
1	D1a	7	4.7–27.6
2	D1b	8	5.16–18.0
3	N	12	5.6–17.9
4	C1	15	6.8–31.0
5	A1	10	4.6–23.0
6	C2	19	4.8–27.6
7	M	14	4.0–27.1
8	A2	10	5.0–12.1
9	K	17	2.35–24.4
10	O	9	6.0–21.0
11	B1	9	3.0–15.7
12	H	8	3.0–32.0
13	F	14	6.0–18.1
14	B2	12	5.0–19.0
15	E	18	5.65–24.0
16	J	3	7.6
17	D2	10	6.64–26.0
18	G	16	2.89–20.1
19	L	13	4.0–27.0
20	I	25	7.0–65.0

Lestari et al. (2013) aligned repeated genomic regions associated with the soybean protein content and identified 35 and 19 candidate genes on chromosomes 10 and 20, respectively. Yang et al. (2019) mapped a protein content-related gene within a 329-kb region on chromosome 15 and predicted that the *Glyma.15g049200* gene might be related to the soybean protein content. In addition. Bolon et al. (2010) mapped a protein content-related QTL in an 8.4 Mb interval between *Sat_174* (24.54 Mb) and *ssrqtl_38* (32.92 Mb) on chromosome 20, and Hwang et al. (2014) further narrowed the region to 2.4 Mb between 27.6 and 30.0 Mb through association analysis and screened six candidate genes (*Glyma20g19680*, *Glyma20g21030*, *Glyma20g21080*, *Glyma20g19620*, *Glyma20g19630*, *Glyma20g21040*). Valliyodan et al. (2016) analyzed 106 soybean lines through re-sequencing and found three gene clusters related to the protein content in a 2.4 MB interval on chromosome 20, in which *Glyma20g19680*, *Glyma20g21030*, and *Glyma20g21080* were further identified as the candidate genes. Vaughn et al. (2014) also identified a protein content-related QTL located about 1 Mb downstream from the region identified by Hwang, and the location of the most significant SNP site was 31,972,955 bp. A total of 13 candidate genes associated with seed protein were detected, 8 of which were highly expressed in mature soybean seeds (Huang et al. 2018). Fliege et al. (2022) conducted fine mapping of chromosome 20 and cloned the major gene *Glyma.20g85100* that controls the protein content, which provides a detailed insights into the haplotype analysis of Glyma.20G085100 for variations in protein content. Analysis of transgenic plants indicated that the regulatory effect of the *cqSeed protein-003* QTL on the protein content was caused by a transposon insertion in the CCT domain encoded by the *Glyma.20G85100* gene. Qin et al. (2022) reported that seven candidate genes associated with seed protein content were identified in a 471-kb haplotype block from Chr6_18844283 to Chr6_19315351, including polynucleotidyl transferase (Glyma.06G202900 and Glyma.06G203100), polygalacturonase activity (Glyma.06G202600 and Glyma.06G203000), ATP synthase (Glyma.06G203200), and genes without annotation (Glyma.06G202700 and Glyma.06G202800). The discovery of more soybean protein content genes is an urgent need for promoting the breeding of new soybean varieties with high protein contents.

Genes related to the oil content may also be involved in protein synthesis and metabolism. For example, phosphoenolpyruvate carboxylase (PEPC) is involved in the dual regulation of both protein and fatty acid synthesis in seeds. Inhibition of the expression of the endogenous *GmPEPC* gene in soybean promoted the accumulation of oil, while the increase in PEPC activity favored the synthesis of protein (Zhang et al. 2017; Zhao et al. 2006). Transcription factors *GmDof4* and *GmDof11* increased the oil content in grains by activating the expression of fatty acid synthesis-related genes such as acetyl-CoA carboxylase gene and by inhibiting the expression of protein synthesis-related genes (Wang et al. 2007; Sun et al. 2018). The *ABI3* (*Abscisic acid insensitive 3*) gene is involved in regulating the synthetic pathways of both protein and oil (Lazarova et al. 2002). Transgenic soybean lines expressing the *Arabidopsis* QQS (Qua-Quine Starch) gene showed 8–10% increase in seed protein content by regulating metabolic processes affecting the partitioning of carbon and nitrogen among proteins and carbohydrates (Li et al. 2015). Furthermore, some soybean sugar transporters such as *GmSWEET10a*, *GmSWEET10b* and *GmSWEET39* had pleiotropic effect on seed protein and oil content (Miao et al.^2020^; Wang et al. 2020b; Zhang et al. 2020). *POWR1* (Protein, Oil, Weight, Regulator 1) gene plays a critical role in controlling the seed quality and yield traits by regulating likely nutrient transport and lipid metabolism, and transgenic study demonstrated that the high-protein *POWR1* allele may be employed to meet the worldwide requirement for high-protein soybean in the breeding process (Goettel et al. 2022).

### Genes related to soybean seed protein components

So far, 625 seed proteins have been identified in soybean seeds and they are classified into 11 groups based on their functions, of which 197 storage proteins are the main components of the proteins in mature soybean seeds (Krishnan et al. 2009; Zhang et al. 2016). Soybean seed storage proteins can be classified into four basic types based on their differences in solubility: albumins, globulins, prolamins, and glutelins (Natarajan et al. 2006). The proportion of each storage protein relative to total protein is affected by both genotype and the environment. The storage proteins of cultivated soybean seeds are mainly composed of 2S, 7S, 11S, and 15S globulin complexes, of which 7S and 11S globulins account for 60–80% of the total seed protein (Singh et al. 2015). 7S globulin (also known as β-conglycinin), which accounts for about 30% of soybean seed protein, is a heterotrimeric glycoprotein that consists of α, α’, and β subunits and has a molecular weight of 126–170 kDa (Hirano 2021; Singh et al. 2015). 11S globulin is a hexameric protein of 320–375 kDa, of which each subunit consists of an acidic (A) and a basic (B) polypeptide chain, accounting for about 40–60% of total seed protein (Tandang-Silvas et al. 2010; Zhao et al. 2021). The proportions of 7S and 11S globulin in soybean seeds are negatively correlated, and the ratio of 11S to 7S (11S/7S) globulin in soybean seed was reported to directly affect the nutritional quality and functional properties of soybean seed protein, thereby affecting the application value of soybean protein (Singh et al. 2015). The content of sulfur-containing amino acids in 11S globulin is 5–6 times that in 7S globulin. A high content of 11S globulin results in higher nutritional quality of soybean protein. Therefore, breeding soybean varieties with high content of 11S globulin can improve the nutritional value of soybean protein and related products (Achouri et al. 2005; Liu et al. 2006; Magni et al. 2018). Furthermore, compared with 11S globulin, 7S globulin has fewer disulfide bonds and a higher lysine content, thus having better emulsifying properties (Fujiwara et al. 2014; Kagawa et al. 1987). Compared to animal proteins, the soybean protein has lower content of sulfur amino acids (Henkel 2000). The sulfur-containing amino acid content in soybean is an important evaluation index of protein quality, however, the limitation of protein quality is the deficiency in sulfur-containing amino acids such as methionine (Met) and cysteine (Cys) (Krishnan et al. 2000). The FAO recommended that the requirement of Cys + Met contents are 3.5% of the total protein (George and de Lumen 1991). In regular soybean varieties, the Met + Cys contents are approximately 2.4% of the total protein (Panthee et al. 2006). Moreover, the value of soybean protein is limited by the Met + Cys contents (2.9g/16gN) (Banaszkiewicz 2011). According to Liebig’s law of the minimum and the concept of the well-known Liebig barrel, Met and Cys are the first and the second limiting amino acid, respectively (Lemme et al. 2020). Therefore, the levels of sulfur-containing amino acids (Met + Cys) are extremely important for soybean. A total of 113 genes encoding for sulfur-containing amino acid enzymes were identified in soybean (https://www.genome.jp/kegg/pathway.html). Panthee et al (2006) identified seven QTLs governing the content of sulfur-containing amino acids using a population of F_6_-derived recombinant inbred lines (RILs), including four (*Satt235*, *Satt252*, *Satt427* and *Satt436*) for Cys and three (*Satt252*, *Satt564* and *Satt590*) for Met concentration. Two QTLs and nine QTLs associated with both Cys and Met content were found in F_5:9_-derived progeny of RILs and 137 Canadian short‑season soybean lines, respectively (Fallen et al. 2013; Malle et al. 2020). The synthesis of sulfur-containing amino acids was regulated by soybean cysteine β-lyase gene *GmCBL1* and its homologous gene *GmCBL2*, and overexpression of *GmCBL1* and *GmCBL2* significantly increased the contents of Met+Cys in transgenic hairy roots. These promising molecular markers and candidate genes may be useful for genetic selection for elevated soybean protein quality. Soybean antigen protein, also known as soybean allergen, may cause allergic reactions in piglets, calves, and fish, which damage the intestinal tract and impede the growth and development of the animals (Sun et al. 2005; Qiang et al. 2018; Li et al. 2021). The allergen database AllergenOnline (http://www.allergenonline.org/) includes 43 soybean allergens, i.e., glycinin, β-conglycinin, Gly m Bd 28 K, Gly m Bd 30 K, soybean hydrophobin (Gly m 1), soybean hull protein, soybean inhibitory protein, and Kunitz trypsin inhibitor (KTI). Glycinin (11S) and β-conglycinin (7S) are the main substances that cause allergy in young animals, and the acidic subunit of 11S globulin can cause allergy in both humans and animals (Adachi et al. 2003). 7S globulin includes lectin, β-amylase, Glym Bd 28 K, Glym Bd 30 K, lipoxygenase, and β-conglycinin. Β-conglycinin is a trimer that consists of α, α’, and β subunits, of which the α and α’ subunits have the highest antigenicity (Fang and Qiu 2005; Amigo-Benavent et al. 2011). 2S globulin also includes a Kunitz trypsin inhibitor, cytochrome c urease, and 2S globulin (Tay et al. 2006). Therefore, determining the contents of 7S and 11S globulin and the proportion of each component in diverse soybean genetic resources and screening elite germplasm resources are of great significance to the improvement of the nutritional and processing quality of soybean protein and the sustainable production and application of protein.

The 7S and 11S storage proteins are encoded by a multigene family (Fischer and Goldberg 1982; Scallon et al. 1985). The 7S globulin gene family has at least 15 members encompassing CG-1 to CG-15 (Krishnan et al. 2000). *CG-1* encodes the α´ subunit, and *CG-4* encodes the β subunit (Harada et al. 1989). Genetic analysis of plant material completely lacking the 7S globulin indicated that this was controlled by a dominant locus, *Scg-1* (Teraishi et al. 2001). The *Scg-1* locus contains the α-subunit gene and β-subunit gene, which are involved in regulating the synthesis of soybean 7S globulin. Silencing both of the genes induced the complete lack of 7S globulin and an increase in the 11S globulin content in soybean (Tsubokura et al. 2012). Overexpression of *OASS* simultaneously increased the content of 7S and 11S globulin, thus increasing the total protein content and contributing to the significant increase in the cysteine and methionine contents (Alaswad et al. 2021). A total of seven genes were found to encode soybean 11S globulin, Gy1–Gy7. The Gy1–Gy5 genes consist of four exons and three introns (Nielsen et al. 1989; Beilinson et al. 2002). Gy1, Gy2, and Gy3 encode A_1a_B_2_, A_2_B_1a_, and A_1b_B_1b_ subunits, respectively, which mainly consist of sulfur-containing amino acids, cysteine, and methionine. Gy4 and Gy5 encode the A_5_A_4_B_3_ and A_3_B_4_ subunits, respectively (Nielsen et al. 1989; Beilinson et al. 2002). The *Gy1* and *Gy2* genes are located on chromosome 3, and they are 3 kb apart and closely linked. *Gy3* is located on chromosome 19 (Nielsen et al. 1989). *Gy4* and *Gy5* are located on chromosomes 10 and 13, respectively (Diers et al. 1992). In addition to the five genes described above, Beilinson et al. also identified two new genes (*Gy6* and *Gy7*). *Gy6* is located downstream of *Gy2* on chromosome 3 and is a pseudogene that cannot encode functional proteins. *Gy7* is in tandem with the 3´ end of *Gy3* on chromosome 19 and has very low expression. Therefore, it is a functional but weakly expressed gene (Beilinson et al. 2002; Nielsen et al. 1989). There are 29 QTLs related to the 7S and 11S globulin contents of soybean storage proteins in the soybean database (www.soybase.org). *Satt461*, *Satt292*, and *Satt156* are in linkage group D2, I, and L, respectively, and are associated with the content of 11S globulin. *Satt461* and *Satt249* are in linkage group D2 and J, respectively, and are associated with the content of 7S globulin (Panthee et al. 2004). Liu et al. (2009) mapped 28 QTLs related to the content of 11S and 7S globulins using a population of recombinant inbred lines. Jian et al. (2013) found 14 SSR sites associated with 11S and 7S globulins through association analysis. Mutagenesis is widely used to induce mutations in the subunits of soybean protein. Zhang et al. (2018) screened six mutants with an obvious mutation in the subunit genes and 10 mutants that had an 11S/7S ratio higher than 3 from EMS-induced mutant lines. Hayashi et al. (2001, 2009) obtained a mutant lacking 7S globulin by means of γ-ray irradiation, and its regulatory genes were mapped on chromosome 19 between markers Satt523 and Sat_388 (3.39 cM) (Hayashi et al. 2001, 2009). From the abundant soybean germplasm resources, studies have screened a variety of elite mutants that lack α, α’, *A3*, (α’ + A4), or (α’ + α) (Liu et al. 2010; Song et al. 2013; Tuo et al. 2014; Zhang et al. 2015). Patil et al. (2017) developed a CGY-2-NIL near-isogenic line with a mutation in the *CGY-2* gene. The creation and identification of these genetic materials, markers, and loci are of great significance in the discovery of the genes that control the quality of the soybean protein and for the genetic improvement of soybean quality and provide a basis for the isolation of soybean genes controlling the 7S and 11S globulin contents and the further application of these genes in soybean breeding and production.

### Genes related to the synthesis and transport of soybean seed protein

Understanding how the molecular mechanisms of synthesis, transport, and storage of seed proteins has great significance for seed quality improvement in soybean (The et al. 2020; Yang et al. 2020). The anabolism of soybean seed protein mainly involves two processes: amino acid synthesis (ammonia assimilation and transport) and the transcription, translation, and processing of storage protein genes (Warsame et al. 2018). The anabolism of soybean seed protein begins with the fixation and conversion of nitrogen (Fabre and Planchon 2000). Soybean converts atmospheric nitrogen into NO_3_^−^ by the action of nitrogenase in rhizobia in a symbiotic nitrogen fixation system formed in soybean roots, which is then converted into NO_2_^−^ by the action of nitrate reductase (NR). Catalyzed by nitrite reductase (NiR), NO_2_^−^ is reduced to NH_4_^+^ (Ohyama et al. 2017). NH_4_^+^ is immediately transported to the cytoplasm of root nodule cells where it is used to synthesize adenine and guanine that are then oxidized and decomposed into ureide. After being transported from roots to hulls, ureide is degraded into glyoxylic acid and ammonium ions (Cabanos et al. 2021). Ammonium ions are converted into glutamine and glutamate through the glutamine synthase–glutamate synthase pathway, and after being activated, they are transported by tRNA to mRNA on the rough surface of the plasma reticulum to synthesize 11S and 7S globulins that are then transported by Golgi receptors to the vacuole for processing and modification. Finally, all storage proteins are combined in a protein body for the synthesis and accumulation of soybean seed protein (Cabanos et al. 2021). Therefore, the seed protein content is derived from the transport of nitrogen compounds (such as amino acids, ureides, peptides) from roots, nodules, and mature leaves (Tegeder and Masclaux-Daubresse 2018).

Some related genes involved in protein endoplasmic reticulum synthesis pathway may cause the significant variations in protein content between Jidou 12 and Ji HJ117 (Guo et al. 2019). Amino acid transporters (AATs) family such as Cationic Amino Acid Transporters (CATs), Amino Acid Permeases (AAPs), and organic nitrogen compounds transporters (NCTs) were identified in recent studies (Cheng et al. 2016; Joaquim et al. 2022). Joaquim et al. (2022) reported that some NCT-related genes *AAP7*, *AVT3*, *CAT9*, *UMAMIT25* and *UPS2* were highly expressed in the high protein content seed, indicating that genetic manipulation of these *NCT* genes may contribute to elevated protein content in soybean seed. The putative amino acid permease transporter gene (*AAP8*, Glyma.08G113400) and seed storage 2S albumin protein gene (Glyma.08G112300) may be responsible for the high water-soluble protein content in soybean. (Zhang et al. 2017).

### The synthesis and transport of storage proteins in soybean seed

During soybean seed development, both 7S and 11S glycinin are initially synthesized on the endoplasmic reticulum (ER) as precursors, and then transported to protein storage vacuoles (PSVs) via the vesicles (Hohl et al. 1996; Vitale and Raikhel 1999). In PSV, most of them are processed into mature subunits and deposited (Müntz 1998). Such as 11S glycinin of soybean, it is first synthesized on the ER as proglycinin, which contains a short signal peptide that directs the precursor transferred to the lumen of the ER, then, the signal sequence is removed and the resultant proglycinin assembles into trimers (Müntz 1998). Proglycinins are sorted to the PSV via the dense vesicle (DV)-mediated post-Golgi trafficking pathway, where they are processed into mature hexameric structure by specific posttranslational cleavage that occurs between Asn and Gly residues (Müntz 1998; Robinson et al. 1998). In addition to the typical “ER-Golgi-PSV” pathway, ER-derived vesicles were observed in β-conglycinin-inhibited transgenic soybean seed, which resembled precursor accumulating vesicles of pumpkin seeds or the protein bodies accumulated in cereal seeds (Hara-Nishimura et al. 1998). Glycinin is a major component of these ER-derived vesicles, thus these ER-derived novel vesicles are called Protein Bodies (PBs) (Kinney et al. 2001). Moreover, the ER-derived precursor-accumulating (PAC) vesicles also involve in the trafficking of Glycinin during the early stage of soybean cotyledon development (Mori et al. 2004). In summary, storage proteins of soybean are transported to the PSV by three pathways: (1) ER-Golgi-PSV, storage proteins are synthesized on the ER as precursors and transported to the PSV through the Golgi by DVs (Robinson et al. 1998). (2) ER-PBs, storage proteins are synthesized on the ER and directly bud from ER to form PBs (Kinney et al. 2001). (3) ER-PAC-PSV, storage proteins are synthesized on the ER and transported to the PSV bypassing the through the Golgi via PAC vesicles (Mori et al. 2004).

In maturing seed cells, vacuolar sorting determinants (VSDs) are required to direct proteins into transport vesicles destined for the PSV. Three kinds of VSD have been identified so far: sequence-specific VSD (ssVSD), C-terminal VSD (ctVSD), and physical structure VSD (psVSD) (Matsuoka and Neuhaus 1999; Vitale and Raikhel 1999). The ssVSDs contain conserved amino acid sequences, such as the NPIR-like motif, which are necessary for recognition by a vacuolar sorting receptor (Koide et al. 1997; Nishizawa et al. 2003). The ssVSDs can be located in N-terminal (e.g., sporamin in sweet potato), C-terminal (e.g., 2S albumin in Brazil nut), or within mature proteins (e.g., 11S glycinin in soybean) (Kirsch et al. 1996; Nishizawa et al. 2006; Saalbach et al. 1996; Vitale and Hinz 2005). ctVSDs are present in C-terminal regions of polypeptides and have highly variable sequence, they are often enriched in hydrophobic amino acids (Neuhaus and Rogers 1998; Nishizawa et al. 2003). In contrast to the ssVSD and ctVSD, there is limited information on the psVSD, which is postulated to depend on the integrity of long internal sequence stretches (Neuhaus and Rogers 1998). A transient expression assay in developing soybean cotyledons demonstrated that the C-terminal 10 residues of the β-conglycinin α′- and β-subunits (PLSSILRAFY; PFPSILGALY; CT10) were shown to act as a necessary and sufficient vacuolar targeting signals in soybean cotyledon cells (Nishizawa et al. 2003). In contrast to β-conglycinin, three types of VSD may coexist in 11S glycinin of soybean (Nishizawa et al. 2006), suggesting that plants have evolved complicated sorting mechanism.

## Study of the soybean seed protein content

### Transcriptomics

High-throughput sequencing-based transcriptomics and proteomics are effective methods to analyze changes in genes and their products in the biological processes of organisms (Lan et al. 2012). Studies show that the soybean seed protein content-related genes are transcriptionally activated and then repressed during embryogenesis, while genes encoding mRNA are transcribed at a similar rate. In the absence of DNA methylation, both transcription and post-transcription processes regulate the mRNA levels of genes that encode soybean seed protein, providing information for improving the soybean seed protein content (Walling et al. 1986). Song et al. (2016) found a large number of differentially expressed genes between a near-isogenic line (cgy-2-NIL) lacking the allergenic α subunit of β-conglycinin and its recurrent parent. The *cgy-2* allele is derived from a functional allele that is closely related to the amino acid quality. The β subunit of soybean seed storage protein is of great importance to the balance of sulfur-containing amino acids and their processing quality. Zhang et al. (2019) found that sulfur-containing amino acids (Cys + Met) were significantly increased (31.5%) in soybean seeds with a low content of the β subunit, implying a close relationship between the β subunit and sulfur assimilation, which work together to coordinate the synthesis of soybean seed protein. In production, the protein content of variety Ji HJ117 (52.99%) was higher than that of the control variety Jidou 12 (46.48%), and the variation may be caused by the difference in protein synthetic pathways that take place in the endoplasmic reticulum during seed development (Guo et al. 2019).

### Metabolomics

Metabolomics is an important method to elucidate the regulatory mechanism of metabolism related to soybean seed growth and the synthesis and degradation of proteins. Lin et al. (2014) identified 169 metabolites in the seeds of 29 conventional soybean varieties and found that the level of 104 metabolites varied significantly among the varieties. At the same time, metabolite markers that can be used to distinguish genetically related soybean varieties were identified, and the results provide a genetic basis for further analysis of soybean seed metabolites. Schmidt et al. (2011) systematically analyzed the soybean SP2 mutant (seed storage protein knockout mutant) using proteomic, metabolomic, and transcriptomic approaches and found that the rebalancing of the seed protein content was largely due to the selective accumulation of a small number of proteins, while the rebalancing of protein components was accompanied by only slight transcriptomic and metabolomic changes.

### Proteomics

Proteins are the final products of gene expression and the specific executors of gene functions. Changes at the proteomic level can directly reflect changes in the execution of gene functions during the growth and development of organisms (Li et al. 2019a). Although the genomic differences between wild and cultivated soybeans have been extensively studied and gradually clarified, differences in their seed protein expression have not been determined. Li et al. (2007) analyzed the differences in seed storage proteins between three wild soybeans and three cultivated soybeans, which provided important information for identifying important specific genes in wild and cultivated soybeans. Hashiguchi et al. (2020) identified 65 proteins that were differentially expressed between wild and cultivated soybean seeds, which may promote the use of wild relatives in transgenic breeding to improve soybean protein and other agronomic traits. Glycinin subunits are believed to have an important role in soybean breeding and the improvement of the biochemical properties of soybean proteins. Cho et al. (2014) identified 10 glycinins from the cotyledon tissue of the soybean seed coat, which provides a theoretical basis for clarifying the genetic regulation of glycinin expression in seeds. Min et al. (2015) found that the urea cycle might be involved in the accumulation of glycinin and β-conglycinin subunits (SSPs), thereby increasing the protein content of soybean seeds. The protein content of a soybean mutant induced by fast neutron (FN) irradiation was increased by 15%. Islam et al. (2020) compared the difference in seed protein expression between the wild-type and FN-induced mutant and found that the level of basic 7S globulin in the mutant was fourfold higher than that in wild-type soybean seeds. This study provides a valuable germplasm resource for clarifying the molecular mechanism regulating the synthesis and degradation of soybean protein. The protein profiles of the high-oil variety Jiyu 73 (JY73) and high-protein variety Zhonghuang 13 (ZH13) were compared, and it was found that the high protein content of ZH13 was mainly due to the high expression of major storage proteins and the proteins related to nitrogen and carbon metabolism (Xu et al. 2015).

## Creation of soybean germplasm with high protein content and the breeding of new varieties

### Integration of multiple breeding approaches accelerates the discovery and utilization of soybean genetic resources with high protein content

There are two major ways to genetically improve soybean protein: one is the use of conventional breeding methods to improve the soybean protein content or protein quality (Tian et al. 2021; Hao et al. 2021; Zhang et al. 2021); the other way is to modify specific genes to achieve the targeted improvement of soybean protein (Lin et al. 2010; Wu et al. 2021; Zhang et al. 2021). Hybridization and mutation breeding are the two major techniques methods used in the breeding of soybean varieties with high protein content, and hybridization is the most commonly used technique to generate variation. According to the genetic characteristics of soybean protein traits, parents with high protein content are selected for hybridization with high-yielding parents to combine the desired traits of both parents, and then new soybean varieties with high protein content and high yield are selected among the progenies. This is the most conventional approach used in the breeding of soybean varieties with high protein content. Mutation breeding for improving soybean quality began in the mid-1970s. The improvement of soybean protein content and quality through physical and chemical mutagenesis is an effective approach for improving soybean quality, which mainly includes chemical, physical, and spatial mutagenesis. The soybean variety Yumeminori with null α´, α subunits and low allergenicity was isolated from the γ-ray irradiation progeny of Kari-kei 434 (Takahashi et al. 1994, 2004), and a four bp insertion mutation in the exon of *CG-2* gene was responsible for the absence of α subunit in Yumeminori (Ishikawa et al. 2006). Guo et al. (2005) used physical mutagens such as ^60^Co-γ rays and thermal neutron irradiation as well as chemical mutagens such as EMS and sodium azide to treat soybean and successively bred the vegetable soybean variety Huaihadou 1 (protein content of 44.93%), the high-protein mutant lines 903,525 (47.60%), 903,526 (47.02%), and 903,527 (47.53%), which have also high resistance to viral diseases and gray spot disease, and two high-protein stable mutants, 923,725 (45.38%) and 923,738 (45.24%). Wei et al. (2019) screened nine stable germplasms (m1–m9) with high protein content among the mutant lines of EMS-treated Zhongpin 661 in the M_7_ generation, and their average protein content was 48.17%, which is 16.94% higher than the protein content (41.19%) of the wild type. Besides, Wang et al. (2021b) screened a new soybean line Nanxiadou 25 from the ^60^Coγ radiation-induced mutant lines of Rongxiandongdou, and Nanxiadou 25 was high protein (50.1%), good shading tolerance, and strong lodging tolerance soybean variety widely cultivated in southwest China. A range of high-protein materials obtained through mutagenesis has enriched the soybean gene pool. Recurrent selection is an effective method to synergistically improve the protein content and yield. Without selective backcrossing for the protein content, the protein content of the backcrossed population generally decreases, and selective backcrossing using material with high protein content can effectively increase the protein content of the population (Zhao et al. 2006).

Combining molecular biological technology with conventional breeding approaches may effectively shorten the breeding time and improve the efficiency of selection in crop quality improvement (Jun et al. 2007). The development of markers related to the soybean protein content such as SSR marker satt182, satt419, satt239, and satt598 has made selection more efficient (Li et al. 2020). The development of high-throughput and low-cost sequencing technologies, chips, and genome-wide selection have improved the selection efficiency in the genetic improvement of the soybean protein content. Increasing the training set size and improving the relationship between the training and validation sets were reported to improve the efficiency in selecting varieties with high protein content, and the predictive ability reached *R*^2^ = 0.81 (Stewart-Brown et al. 2019). The selection of a representative and diverse training population increased the predictive ability related to the protein content to 0.92 (Jarquin et al. 2016). For a training population with fewer than 350 individuals, the predictive ability of the protein content increased with the training population size, and the prediction accuracy increased significantly with the number of markers when fewer than 1000 markers were used in the model (Beche et al. 2021). The use of intragenic markers in predicting the protein content increased the predictive ability by 0.02 compared with the use of intergenic markers, and the addition of progeny lines to the training population established with germplasm resources increased the predictive ability from 0.47 to 0.65 (Sun et al. 2022). In addition, the inclusion of epistatic effects in the model also improved the accuracy of predicting the protein content (Duhnen et al. 2017). With the advancement of sequencing technology and an in-depth understanding of the factors that contribute to the prediction accuracy, genome-wide selection is expected to display superiority in the selection of multi-gene-controlled complex traits including the protein content. The cloning of protein content-related genes is conducive to the targeted modification of specific genes and the improvement of soybean protein. The content of sulfur-containing amino acids in soybean is affected by genes, the environment, and the interaction between genes and the environment. Traditional breeding approaches have low selection efficiency and slow progress. The use of molecular biological methods is conducive to improving the efficiency of selecting for the amino acid content. Dinkins et al. (2001) transformed a 15 kDa δ-zein gene into soybean using particle bombardment, and in the transgenic plants, the content of methionine was increased by 12–20% and the content of cysteine was increased by 15–35%. In the same way, Li et al. (2005) obtained transgenic soybean plants with a 27 kDa γ-zein gene, in which the content of methionine was increased by 15.49–18.57% and the content of cysteine was increased by 26.97–29.33% compared with control plants. A proglycinin gene *Gy1* (A1aB1b) with a synthetic DNA encoding four continuous methionines (*V3-1*) was connected between the *hpt* gene and the modified green fluorescent protein sGFP (S65T) gene, and a resultant plasmid was introduced into soybean plants using particle bombardment. The result showed that compared to control plants, transgenic soybean plants accumulated higher levels of glycinin (El-Shemy et al. 2007). The soybean resources with high protein content and high-quality proteins have greatly benefited sustainable protein production.

### Utilization of wild soybean in the breeding of varieties with high protein content

The use of elite germplasm or genes of wild soybean to broaden the genetic basis of cultivated soybean is conducive to the breeding of new soybean varieties with high yield, high quality, and high protein content. Using cultivated soybean Heinong 35 and wild soybean ZYD 355 and ZYD665 as parents, Lai et al. (2005) bred a new soybean line, Longpin 8807, with protein content higher than 48.29%. Du and Yu (2010) crossed the cultivated soybean N23674 (protein content 42.49%) with wild soybean BB52 (protein content 40.09%) that grew naturally in a tidal flat area and screened the F_4:5_ line 4035 that had a distinctly superior quality: a high protein content (49.13%); increases of 0.03–0.76% in the contents of the essential amino acids threonine, lysine, histidine, phenylalanine, leucine, isoleucine, and valine compared with the two parents; and the sulfur-containing amino acid content was the same as that of the wild parent and 0.28% higher than that of the cultivated soybean parent. Eickholt et al. (2019) crossed wild soybean PI 366122 with cultivated soybean variety N7103 and selected eight new breeding lines with high protein content. The protein content of the lines was on average 0.4–4.0% higher than that of N7103 (43.7%), and the highest was 47.7%. The availability of varieties and lines with high protein content developed using wild soybeans has facilitated the breeding of new soybean varieties with high quality, high yield, and a high protein content and has promoted the development of the soybean industry to effectively secure the sustainable and stable supply of protein.

### Utilization of cultivated soybean in the breeding of varieties with high protein content

**Fig. 3:**
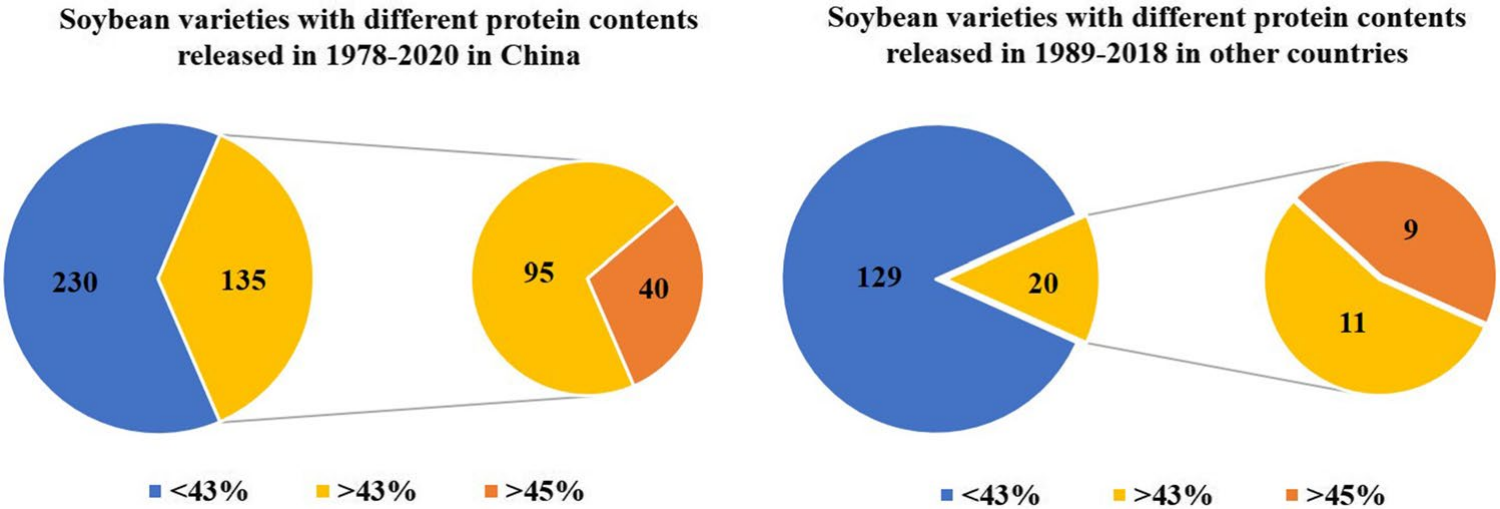
The contribution of soybean varieties with different protein contents released in the world

Many efforts have been made to genetically improve the protein content in soybean. The choice of the most suitable parents is crucial in improving the soybean protein content using conventional breeding approaches, and the principle of parental matching depends on the heritability of the soybean protein content and gene action. The protein content of hybrid progenies is closely correlated with the protein content of the parents (Liu et al. 2016). The protein content of hybrid progenies tends to be stable in the F_4_ generation, and at this time, the selection efficiency is higher. The protein content of hybrid progenies can be directly affected by that of their male parent if they have a female parent with high protein content (Wang et al. 2013). Elevated seed protein level in high yielding varieties should not be at the expense of seed numbers (Rotundo et al. 2009). In the breeding of soybean varieties with high protein content, the correlation between the protein content and other agronomic traits in hybrid progenies needs to be considered. In particular, the improvement of the protein content, 100-grain weight, and yield needs to be synergistic, which forms the basis for the breeding of new soybean varieties with high yield and high protein content (Chen et al. 2002; Wang et al. 2021a, b). Moreover, protein content needs to be prioritized over yield and other agronomic traits, and breeding strategies also need to be further adjusted to meet protein requirements in harvested soybeans (https://germination.ca/is-high-protein-high-yielding-soy-possible/). High protein soybean means the increased protein amount for animal feed, and a single percentage point increase in the protein content will represent millions of tons of additional protein (https://www.syngenta.ca/market-news/new-research-could-increase-soybean-protein-content). The Bayer breeding company have attempted to balance protein, yield, herbicide tolerance and disease resistance for global food security, and the objective of its soybean program project was to develop elite commercial soybean products with an increase in seed protein while maintaining yield and agronomic traits (https://germination.ca/is-high-protein-high-yielding-soy-possible/). In Canadian specific food-grade soybean program, more efforts were made to develop the leading high-protein varieties with higher soybean protein and minimal impacts on yield for the soybean markets (https://germination.ca/is-high-protein-high-yielding-soy-possible/). From 1978 to 2020, a total of 135 soybean varieties had the protein content higher than 43% in China, of which 40 soybean varieties had higher protein content (> 45%) (Fig. 3). From 2003 to 2016, a total of 251 soybean varieties were approved in China, of which 17.27% had a protein content higher than 43% (Liu et al. 2017). From 1989 to 2018, a total of 20 soybean varieties had the protein content higher than 43% in other countries, while only 9 soybean varieties had higher protein content (> 45%) (Fig. 3). A series of diversified soybean germplasms with higher protein content (> 45%) were bred through hybridization in the world, such as X3585-116-3-B (50.1%), Nandou12 (51.79%), NC 104 (50.7%), BARC-9 (52.9%), D90-7256 (50.5%), Hipro (53.9%) and Saedanbaek (48.2%) (Table 2). Many soybean varieties had the significant higher content for seed protein in China, such as 903,525 (47.60%), 903,526 (47.02%), Guichundou108 (47.86%), Zhechun 4 (47.96%), Fudou 234 (47.88%) and Nandou12 (51.79%) (Table 2). For the United States, conventional breeding had been used to generate some high protein soybean varieties, including NC 104 (50.70%), BARC-8 (52.80%), BARC-9 (52.90%), D90-7256 (50.50%) in 1986–2020 (Table 2). Besides, Lee (PI548656) and Harosoy (PI548573) were important breeding materials in United States (https://soybase.org/uniformtrial/index.php?filter=+Essex+&page=lines&test=ALL). AC Proteus (52.10%), AC Proteina (49.80%), X3585-116-3-B (50.10%), HS-151 (46.70%), HS-161 (46.20%) and HS-162 (48.10%) were the high protein varieties that were identified and reported in Canada (Table 2). Ontario was the main soybean-producing province in Canada, and the Ontario soybean variety trials in 2005–2021 were conducted by the Ontario Soybean and Canola Committee (www.Gosoy.ca). In 2021, three soybean varieties Primo (48.4%), BAFFIN (48.5%) and Atiron (48.7%) exhibited higher protein content than most soybean varieties in the Ontario soybean variety trial. Besides, more soybean varieties with high protein content are accessible in the Canadian food-grade soybean variety database (https://buycanadiansoybeans.ca/food-grade soybeans/). Greya (46.30%), Saedanbaek (48.20%), and Hipro (53.90%) were the high protein varieties in Russia, Korea, and Republic of Korea, respectively (Table 2).

**Table 2:** List of high-protein (> 45%) soybean germplasms developed by conventional breeding

Country	Germplasm	Protein content (%)	Pedigree	Reference
China	903,525	47.60	Hefeng 22/PI407788A	Guo et al. (2005)
China	903,526	47.02	Hefeng 22/PI407788A	Guo et al. (2005)
China	Xingdou 5	45.90	Kefeng 6/Yudou 28	Zhu et al. (2008)
China	Huaxia 4	46.15	Guizao 1/Brazil 8	Ouyang et al. (2009)
China	Huachun 6	45.80	Guizao 1/Brazil 8	Li (2010)
China	Gongqiudou 5	45.46	Gongxuan 1/Gongqiudou 3	Fan et al. (2016)
China	Meng 1301	45.26	Hedou 3/Fudou 9	Zhang et al. (2019)
China	Guichundou108	47.86	Quandou 937/Guizao 1	Yang et al. (2020)
China	Mudou 15	45.08	Heinong 48/Longpin8807	Wang et al. (2020c)
China	Xudou 25	45.49	Xudou 9/Yushandadou	Zhou et al. (2021)
China	Zhonglongdou 106	45.96	Heinong48/Wuxing 4	Liu et al. (2021)
China	Nannong 49	46.84	Xiangchundou 26/Taiwan 292	https://www.chinaseed114.com/
China	Huaxia 14	45.40	Guixiadou 2/Nandou 12	https://www.chinaseed114.com/
China	Xudou 23	45.22	Xudou 9/Zheng 90,007	https://www.chinaseed114.com/
China	Huachun 11	46.73	Huachun 3/Fudou 310	https://www.chinaseed114.com/
China	Shengdou 32	45.69	Xudou 18/Hedou 12	https://www.chinaseed114.com/
China	Nandou 12	51.79	Chengdou 4/Gongdou 6	https://www.chinaseed114.com/
China	Quqiu 6	46.00	Zheqiudou 3/Maoqiupeng	https://www.chinaseed114.com/
China	Shengdou 40	45.61	Huachun 3/Fudou 234	https://www.chinaseed114.com/
China	Wandou 39	45.81	Hedou 3/Jidou 12	https://www.chinaseed114.com/
China	Zhechun 4	47.96	Wuxing-5/Zhechun3	https://www.chinaseed114.com/
China	Fudou 234	47.88	Pudou8008/Huangshadou	https://www.chinaseed114.com/
America	NC 104	50.70	D55-4110/N56–4071	Carter et al. (1986)
America	NC 105	48.70	D55-4110/N56–4071	Carter et al. (1986)
America	NC 106	50.40	D55-4110/N56–4071	Carter et al. (1986)
America	BARC-7	49.10	CX797-21/D80-6931	Leffel (1992)
America	BARC-8	52.80	CX797-21/NC-2–62	Leffel (1992)
America	BARC-9	52.90	CX797-21/NC-2–62	Leffel (1992)
America	D90-7256	50.50	Forrest/D76-8070	Hartwig (1996)
America	Prolina	46.10	Complex hybridization	Burton and Wilson (1999)
America	DMK93-9048	46.20	D86-3429/Braxton	Kenty et al. (2001)
America	R95-1705	46.70	Hutcheson/BARC-7	Chen et al. (2008)
America	N6202	45.70	N6201/N95-7390	Carter et al. (2010)
America	R05-1415	46.90	MFS-591/V96-4486	Chen et al. (2011)
America	R05-1772	46.10	R95-1705/V96-4181	Chen et al. (2011)
America	UA 5814HP	45.40	R95-1705/S00-9980–22	Chen et al. (2017)
America	X3144-48–1-B	46.90	AC Proteus/Maple Glen	Samanfar et al. (2019)
America	TN15-4009	46.00	TN09-016/S05-11,482	Pantalone and Wyman (2020)
Canada	AC Proteus	52.10	Complex hybridization	Voldeng et al. (1996a)
Canada	Maple Glen	45.70	Complex hybridization	Voldeng et al. (1996b)
Canada	AC Proteina	49.80	Complex hybridization	Cober and Voldeng (2000)
Canada	X3585-116–3-B	50.10	Maple Glen/AC Proteus	Cober and Voldeng (2000)
Canada	HS-151	46.70	Complex hybridization	Yu et al. (2016a)
Canada	HS-161	46.20	Complex hybridization	Yu et al. (2016b)
Canada	HS-162	48.10	Complex hybridization	Yu et al. (2016c)
Canada	HS-182	45.70	Complex hybridization	Yu et al. (2019a)
Canada	AAC Wigle	45.80	RCAT0606SCN/SG01-0217EMMM-1	Yu et al. (2019b)
Russia	Greya	46.30	Vilana/Valenta	Zelentsov et al. (2020)
Korea	Saedanbaek	48.20	MD87L/SS92414	Kim et al. (2014)
Republic of Korea	Hipro	53.90	Saedanbaek/Daepung	Kim et al. (2021c)

Mutagenesis is an effective means to increase the protein content of soybean. After the ethyl methanesulfonate (EMS) treatment of Jidou 1 and Jidou 6 seeds, three mutant lines with high protein content were selected in the M_4_ generation (Yu et al. 1995). A batch of soybean varieties with a high protein content such as Heinong 41 (protein content 45.23%) and 90-3527 (protein content 47.53%) was successively bred using γ-ray and thermal neutron irradiation as well as chemical mutagens such as EMS and sodium azide (NaN_3_) (Wang et al. 2000). Wei et al. (2019) reported that continuous selection of plants with a high protein content in the M_2_ generation resulted in an overall decrease in the protein content. In contrast, continuous selection of plants with a low protein content in the M_2_ generation resulted in distinct genetic gain. Gene silencing (Herman et al. 2003) and gene editing technologies (Sugano et al. 2020) have been successfully used to reduce soybean allergenic proteins. Soybean varieties with a high protein content and high quality such as Qihuang 34 and Kexin 3 were bred through the combination of mutation and hybridization techniques. Soybean varieties and mutant lines were also developed by means of ^60^Co γ-ray and thermal neutron irradiation and treatment using the chemical mutagens EMS and sodium azide, which include vegetable soybean variety Huaihadou 1 (protein content 44.93%), the three high-protein mutant lines 903,525 (47.60%), 903,526 (47.02%), and 903,527 (47.53%) that mature early and have high resistance to viral diseases and gray spot, and two high-protein stable mutants, 923,725 (45.38%) and 923,738 (45.24%) (Guo et al. 2005). Wei et al. (2019) screened nine stable germplasms (m1–m9) from the M_7_ generation of EMS-treated Zhongpin 661, and they had an average protein content of 48.17%, which is 6.98% higher than that of the wild type (41.19%). Those high-protein resources have greatly contributed to the sustainable production of protein.

With regard to the productive efficiency, protein yield per unit area should be taken seriously in practical breeding process. Protein yield is calculated by multiplying yield and protein content. The high positive correlation relationship was found between protein yield and yield, however, the low correlation relationship was found between protein yield and protein content (Kurasch et al. 2017b). Besides, the dense-tolerant trait is a good agronomic trait for the improvement of yield and further protein yield in soybean. Dense-tolerant planting genotype can be introduced to achieve the more self-sufficient in protein yield. Europe depended heavily on soybean imports to meet the growing demand for plant protein, especially for animal feed (https://insights.figlobal.com/plant-based/european-soy-quest-protein-self-sufficiency). There is an urgent need to solve the soybean protein shortage and improve the protein self-sufficiency in Europe. Food-grade soybean is generally used for production of soy milk, tofu and other soy-based food (Jegadeesan and Yu 2020). Kurasch et al. (2017b) revealed that high-yield soybean variety appears to be the powerful strategy for achieving the high protein yield for animal feed, while high protein soybean variety appears to be beneficial for food-grade soybean production in Europe. Coupling high yields with high protein is the Holy Grail in practical breeding process (https://germination.ca/is-high-protein-high-yielding-soy-possible/). Nevertheless, how to balance it well is of great importance for soybean production.

### Breeding soybean varieties with high-quality protein for forage

Soybean is an important source of protein for forage, which is not only rich in nutrients but also contains allergenic proteins and protease inhibitors that prevent the animals from digesting and absorbing proteins. Screening germplasm resources that lack allergenic proteins and utilizing conventional hybridization integrated with modern molecular technology are important strategies to bread high-quality soybean varieties for forage. About 80% of Japanese soybeans lack the allergenic protein Gly m Bd 28 K (Ogawa et al. 2000). Guan et al. (2004) found one accession lacking the β subunit and 77 accessions lacking the allergenic protein 28 K among 175 accessions of Chinese soybean germplasm resources. Soybean Kunitz trypsin inhibitor is a common anti-nutritional factor. Jiang et al. (2020) used conventional hybridization aided by molecular marker-assisted selection employing non-denaturing polyacrylamide gel electrophoresis and selected a new soybean line HZ8009 without the Kunitz trypsin inhibitor. Phytic acid (phytate) benefits the normal growth and development of soybean plants but is an anti-nutritional factor and cannot be digested and utilized by humans and non-ruminants. Myo-inositol phosphate synthase (MIPS) is involved in the synthesis of phytic acid. Hize et al. (2002) and Yuan et al. (2007) silenced the gene using mutagenesis or a molecular biological approach and developed LR33 and Gm-lpa-TW-1 that have a greatly reduced phytic acid content in the seeds; the seed phytic acid content of Gm-lpa-TW-1 decreased by more than 50% compared with that of the wild type parent. A molecular marker for the LPA trait of Gm-lpa-TW-1 was developed (Yuan et al. 2010). These studies and the creation of genetic materials provide references for the improvement of soybean protein quality and the breeding of soybean varieties for forage utilizing modern biological technologies.

### Breeding multifunctional soybean varieties with different seed storage protein components

Since 7S and 11S globulins had a great impact on the nutrition and quality of soybean products, studies had been carried out to develop soybean varieties with different subunit compositions. An inverse correlation was found between the 7S and 11S globulin contents, and the main allergen was included in the subunit of 7S globulin (Ogawa et al. 1989, 1995). At the early stage, soybean breeding was mainly focused on low content of 7S globulin and high content of 11S globulin in soybean seed. Yumeminori was regarded as a low level of 7S globulin (null α´, α subunits), high level of 11S globulin, high content of sulfur amino acids and low allergenicity soybean variety, which was developed by using the γ-ray irradiation method (Takahashi et al. 2004). Another new soybean variety Nagomimaru with α and α' subunits deficiency was developed from the progeny of cross between Kariko 0542 and Tachinagaha, and Nagomimaru was suitable for making soy milk in Japan (Hajika et al. 2009). Soybean seed with high protein content (> 45%), high 11S/7S ratio and suitable subunit composition of 11S is desirable for tofu production and texture (Jegadeesan and Yu 2020). Moreover, the quality and stability of soymilk is also affected by the subunit compositions (Nik et al. 2009). Poysa et al. (2006) showed that α´ subunit of 7S globulin deficiency, A_4_ subunit of 11S globulin deficiency and A_3_ subunit of 11S globulin played major role in contributing to tofu quality. IWATE-3 introduced from Japan, which lacks the α′ subunit of 7S glycinin and all the 11S glycinin, was used for elite soybean germplasm in Canadian soybean breeding (Zarkadas et al. 2007; Yu et al. 2016b, c). Additionally, a wild soybean lacking all three α, α', and β subunits of 7S globulin was a valuable genetic resource for soybean breeding (Hajika et al. 1996, 1998). Two high protein soybean varieties HS-161 lacking the α' subunit of 7S globulin and A_3_ subunit of 11S globulin and HS-162 lacking the α' subunit of 7S globulin and A_4_ subunit of 11S globulin are suitable for soybean food production (Yu et al. 2016b, c). Particularly, HS-162 had excellent processing quality for tofu and soy milk, and HS-162 made firmer tofu than the check tofu-type soybean variety Harovinton (44.90% of seed-protein content) (Buzzell et al. 1991; Yu et al. 2016c). It was reported that 7S globulin can improve lipid metabolism (Mochizuki et al. 2009). The soybean variety Nanahomare contains approximately 1.8-fold 7S globulin than ordinary varieties, but lacks all the 11S globulin (Yagasaki et al. 2010). Nishimura et al. (2016) revealed that 7S globulin-rich soybean variety Nanahomare played a crucial role in decreasing the serum triglyceride level. Nanahomare might be used as a potential soybean resource for 7S globulin-rich soybean breeding and provide the required amounts of 7S globulin for prevention of lifestyle-related diseases such as high serum triglyceride. These soybean varieties with modified subunits can be good genetic resources for improvement of nutrient content and functional components.

## Future expectations

### Clarification of the genetic basis for the formation of protein traits

Soybean protein is a complex quantitative trait. The soybean protein content is closely related to the climate. High-rainfall and high-temperature regions such as Tonghua, Jilin Province (Northeast China), Zhengzhou, Henan Province (Huanghuaihai region), and southern China have mostly favorable conditions for the production of a high protein content. The soybean growing regions were established by the Department of Plant Industry Management of the Ministry of Agriculture and Rural Affairs in 2003, and the set standard for high-protein soybean is 43% for northern regions and 45% for Huanghuaihai and the southern regions. The soybean protein content is negatively correlated with other traits such as yield and the oil content, which cause difficulties in the breeding of high-protein soybean. In order to break the linkage between the protein content and other traits including yield and the oil content, it is necessary to identify the genes related to the formation of soybean protein on a genome-wide scale. In particular, it is necessary to discover the genes that cause the stable expression of a high protein content. For example, soybean variety Jinghe 1 has a high protein content (higher than 48%) in Heihe city, Heilongjiang Province, China. It has extraordinary environmental stability and a high protein content in high latitude regions. Therefore, it is an important resource for discovering genes related to a high protein content. The genetic loci associated with the soybean protein content, key genes and regulatory mechanisms involved in the synergistic regulation of the soybean protein content and yield, as well as the genetic basis for the formation of soybean protein and the key molecular modules or regulatory networks for design breeding can be systematically and precisely elucidated based on the available germplasm resources, large amount of data, as well as technical means in population genetics, genomics, systems biology, and bioinformatics.

### Systematic identification of genetic variation in genes related to the protein content

The essence of breeding is to utilize the association between genotype and phenotype to screen useful genes or allelic variations to obtain varieties with desired comprehensive traits. The soybean protein content is mainly regulated by multiple genes rather than a single gene. Pyramiding multiple desired genes or allelic loci is the strategy used in soybean breeding for a high protein content. Therefore, examining the genetic variation of genes related to the soybean protein content in diverse germplasm resources and their correlation with phenotypes and the screening of germplasm with excellent allelic variation are the keys to improving the efficiency of breeding soybean varieties with high protein content. The availability of cultivated and wild soybean reference genomes as well as functional chips such as SoySNP618K (Li et al. 2022) and ZDX1 (Sun et al. 2022) facilitate the re-sequencing or microarray-based sequence analysis of germplasm resources. Large-scale processing of massive amounts of data obtained from sequencing or microarray analysis improves the efficiency and accuracy of detecting genetic variation, which can be combined with the analysis of phenotypic data of the soybean protein content obtained from various growing conditions to identify useful alleles and establish linear or non-linear models predicting the relationship between the genetic variation and protein content, so as to understand the regularity of the interaction between genes related to the protein content and the environment and to discover desirable alleles that adapt to specific environments.

### Molecular design boosts high-protein soybean breeding

The breeding of breakthrough varieties often depends on the utilization of rare and desired resources (Wehrmann et al. 1987). The availability of the soybean reference genome (Schmutz et al. 2010), wild soybean genome (Kim et al. 2010), pan-genome (Li et al. 2014), and graph-based pan-genome (Liu and Tian 2020) is conducive to the discovery of genes related to the protein content of soybean. The utilization of genes related to the protein content of wild soybean can improve the protein content of cultivated soybean. The discovery of QTLs and genes related to the protein content of cultivated soybean can facilitate the breeding of soybean varieties with high protein content by means of transformation and gene-editing technologies (Wu et al. 2021; Valliyodan et al. 2016). With the establishment and improvement of massive amounts of data, the comprehensive use of modern breeding technologies on the basis of bioinformatics and CRISPR/Cas9 has become an important method for plant improvement and germplasm creation (Gao et al. 2021). Li et al. (2019b) designed sgRNAs for nine different main storage protein genes and used CRISPR/Cas9 technology to edit the soybean seed storage protein gene family. The mutations in three storage protein genes were detected in soybean hairy roots, and the mutation frequency ranged between 3.8 and 43.7%. These studies laid a basis for the use of molecular design to boost the breeding of new soybean varieties with high protein content.

**Fig. 4:**
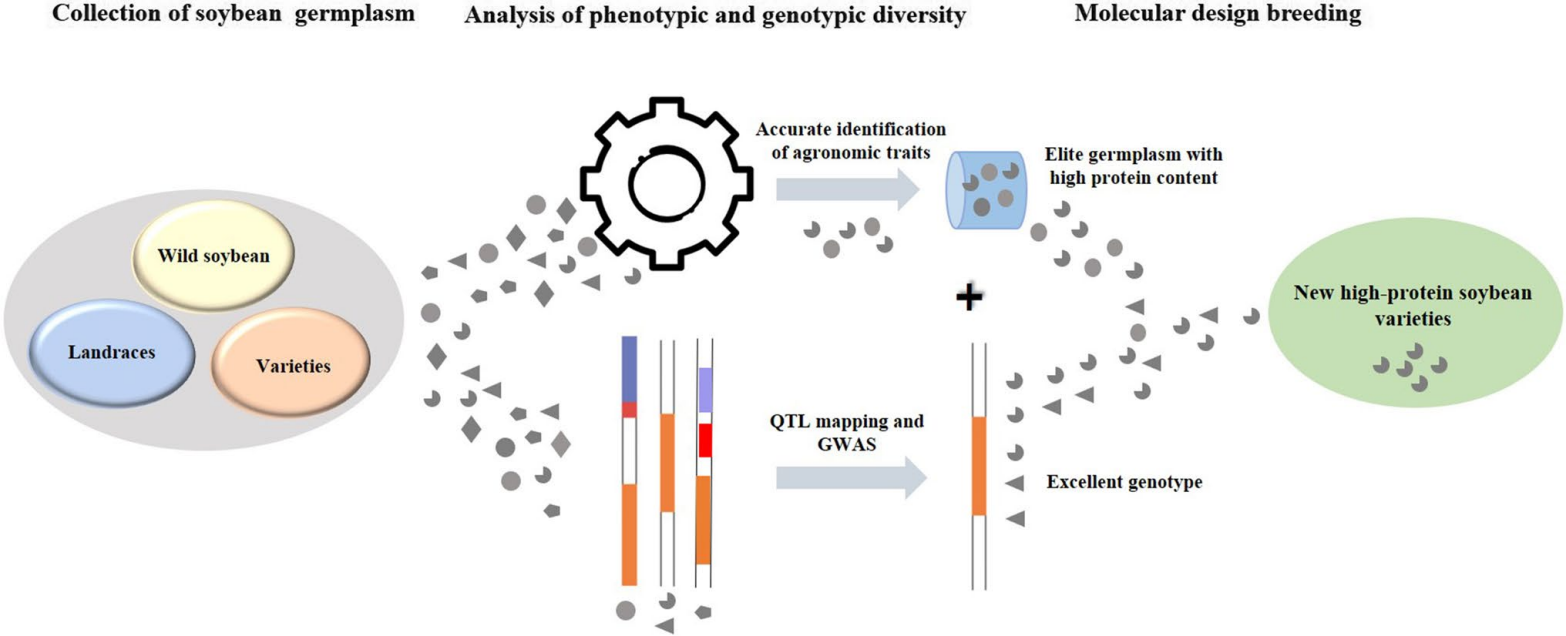
Strategy for breeding of high-protein soybean

It is expected that genes related to the soybean protein content and their genetic variation on a whole-genome scale will be systematically elucidated. The key molecular modules and regulatory networks of genes related to the protein content and the genetic mechanisms of their interactions with yield and the environment will be clarified. In addition, new technologies such as gene editing, gene transformation, synthetic biology, and artificial intelligence are maturing, and under the background of the advancement of above-mentioned technologies, molecular design breeding or intelligent breeding will be applied in the targeted breeding of soybeans with high protein content, high quality, and high yield (Fig. 4), which will benefit the sustainable production of protein and secure the supply of food and dietary nutrients on a global scale.
