# Correction to: Soybean genetic resources contributing to sustainable protein production

**DOI:** 10.1007/s00122-022-04241-6

**Published:** 2022-11-24

**Authors:** Bingfu Guo, Liping Sun, Siqi Jiang, Honglei Ren, Rujian Sun, Zhongyan Wei, Huilong Hong, Xiaoyan Luan, Jun Wang, Xiaobo Wang, Donghe Xu, Wenbin Li, Changhong Guo, Li-Juan Qiu

**Affiliations:** 1 Key Labaratory of Molecular Cytogenetics and Genetic Breeding, College of Life Sciences and Technology, Harbin Normal University, Harbin, China; 2 Nanchang Branch of National Center of Oil Crops Improvement, Jiangxi Province Key Laboratory of Oil Crops Biology, Crops Research Institute of Jiangxi Academy of Agricultural Sciences, Nanchang, China; 3 The National Key Facility for Crop Gene Resources and Genetic Improvement (NFCRI), MOA Key Lab of Soybean Biology (Beijing), Institute of Crop Science, Chinese Academy of Agricultural Sciences, Beijing, China; 4 School of Agronomy, Anhui Agricultural University, Hefei, China; 5 Soybean Research Institute, Heilongjiang Academy of Agricultural Sciences, Harbin, China; 6 College of Agriculture, Yangtze University, Jingzhou, China; 7 Biological Resources and Post-Harvest Division, Japan International Research Center for Agricultural Sciences, Tsukuba, Japan; 8 Soybean Research Institute, Key Laboratory of Soybean Biology of Chinese Education Ministry, Northeast Agriculture University, Harbin, China

**Correction to: Theoretical and Applied Genetics**

10.1007/s00122-022-04222-9

The order of author list has been incorrectly published in the original publication. The complete correct order of author list is given below.

Bingfu Guo^2,3^, Liping Sun^2^, Siqi Jiang^1,3^, Honglei Ren^5^, Rujian Sun^3^, Zhongyan Wei^3^, Huilong Hong^3,8^, Xiaoyan Luan^5^, Jun Wang^6^, Xiaobo Wang^4^, Donghe Xu^7^, Wenbin Li^8^, Changhong Guo^1^, Lijuan Qiu^3^.

The original article has been corrected.

